# E-Cadherin-Coated Plates Maintain Pluripotent ES Cells without Colony Formation

**DOI:** 10.1371/journal.pone.0000015

**Published:** 2006-12-20

**Authors:** Masato Nagaoka, Uichi Koshimizu, Shinsuke Yuasa, Fumiyuki Hattori, Hao Chen, Tomofumi Tanaka, Masaru Okabe, Keiichi Fukuda, Toshihiro Akaike

**Affiliations:** 1 Department of Biomolecular Engineering, Graduate School of Bioscience and Biotechnology, Tokyo Institute of Technology Yokohama, Japan; 2 Daiichi Asubio Pharma Co., Ltd., Biomedical Research Laboratories Osaka, Japan; 3 Department of Regenerative Medicine and Advanced Cardiac Therapeutics, Keio University School of Medicine Tokyo, Japan; 4 Genome Information Research Center, Osaka University Osaka, Japan; Baylor College of Medicine, United States of America

## Abstract

Embryonic stem (ES) cells cultured on gelatin-coated plates or feeder layers form tight aggregated colonies by the E-cadherin-mediated cell-cell adhesions. Here we show that murine ES cells do not make cell-cell contacts or form colonies when cultured on the plate coated with a fusion protein of E-cadherin and IgG Fc domain. The cells in culture retain all ES cell features including pluripotency to differentiate into cells of all three germ layers and germ-line transmission after extended culture. Furthermore, they show a higher proliferative ability, lower dependency on LIF, and higher transfection efficiency than colony-forming conditions. Our results suggest that aggregated colony formation might inhibit diffusion of soluble factors and increase cell-cell communication, which may result in a heterogeneous environment within and between surrounding cells of the colony. This method should enable more efficient and scalable culture of ES cells, an important step towards the clinical application of these cells.

## Introduction

Cell-cell and cell-matrix interactions play a crucial role in migration, proliferation and differentiation during embryonic development [1], [2] and also in the maintenance or regulation of ES cell features. Prudhomme et al. reported that the extracellular matrix might affect the self-renewal of or differentiation signalling in ES cells [3], while E-cadherin, a Ca^2+^-dependent cell-cell adhesion molecule [4], [5], is reported as being essential for intercellular adhesion, colony formation and the differentiation of ES cells [6], [7]. Furthermore, E-cadherin-null mutations in mice are embryonic lethal, because just after compaction, the adhesive cells of the morula dissociate, leading to the failure of preimplantation [8]–[10]. Recent studies have also confirmed that E-cadherin-mediated cell-cell adhesion is frequently rearranged during early embryogenesis to regulate cell migration, cell sorting and tissue formation [11], [12]. Given these findings, we focused on the intercellular adhesion molecule, E-cadherin, and used a fusion protein of the E-cadherin extracellular domain and the IgG Fc domain (E-cad-Fc) [13] as a model matrix for cell adhesion instead of the conventionally used extracellular matrices as a surface-coating material, to clarify the effect of E-cadherin on the maintenance of pluripotency of ES cells.

## Results

### ES cells can adhere onto an E-cadherin-immobilized surface without colony formation

**Figure 1:**
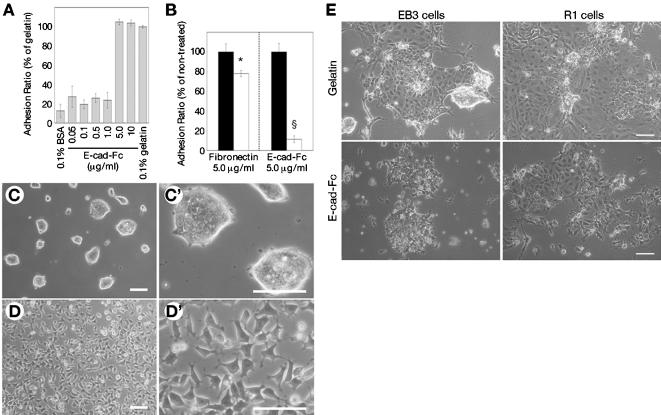
Cell adhesion, morphology of ES cells on the E-cad-Fc fusion protein-immobilized surface. (A) ES cells (EB3) adhered to E-cad-Fc-coated dishes with equivalent efficiency as to 0.1% gelatin-coated dishes after 3 hours of incubation. (B) ES cells (EB3) were cultured on E-cad-Fc-coated or fibronectin-coated dishes without serum. EGTA (5 mM) was added to the culture medium at 3 hours after seeding (open bar). Detached cells were removed and remaining cells were counted using alamar Blue reagent. *:*P*<0.05, §:*P*<0.001 vs. no treated condition (closed bar). (C and D) Morphological observation of ES cells (EB3) on the two different matrices. ES cells were cultured on polystyrene surfaces coated with 0.1% (wt/vol) gelatin (C), or 10 µg/ml E-cad-Fc (D) in the presence of LIF for 2 days. High magnification images are shown in (C′) and (D′). (E) ES cells (EB3 and R1 cells) were cultured on the plates coated with gelatin or E-cad-Fc and differentiation was induced by the withdrawal of LIF. Morphological characteristics were observed as phase contrast images. Bar indicates 100 µm. The data indicate means±SD of 3 separate experiments.

We first tested the ability of two different mouse ES cell lines (EB3 [14] and R1 [15]) to adhere to the E-cad-Fc-coated surface. EB3 cells adhered to the E-cad-Fc-coated surface with the same efficiency as to the conventional gelatin-coated surface, and this adhesion was Ca^2+^-dependent (Figure 1A and B). They adhered even in serum-free conditions, suggesting that adhesion molecules in serum were not the mediators of this adherence to the plates. On the conventional gelatin-coated surface, ES cells formed tightly aggregated colonies (Figure 1C). In contrast, ES cells cultured on the E-cad-Fc-coated surface showed a spindle-like morphology, and were scattered with no evidence of cell-cell contact or colony formation, even in the presence of LIF (Figure 1D). High magnification images demonstrated that the cells remained separated from each other and showed a dendritic morphology with many pseudopodial protrusions (Figure 1D′). Another ES cell line, R1 cells showed the same morphology and Ca^2+^-dependent adhesion to E-cad-Fc as EB3 cells (data not shown). On time-lapse video microscopy, ES cells moved freely in an amoeboid fashion on the E-cad-Fc-coated surface using their pseudopodia (Movies S1 and S2, in Supporting Information). On the gelatin-coated surface, however, the cells formed aggregated masses. Interestingly, differentiated ES cells did form colonies on the E-cad-Fc-coated surface (Figure 1E), suggesting that cell mobility was specific to undifferentiated ES cells.

### ES cells maintain an undifferentiated state and pluripotent ability on the E-cad-Fc-coated surface

**Figure 2:**
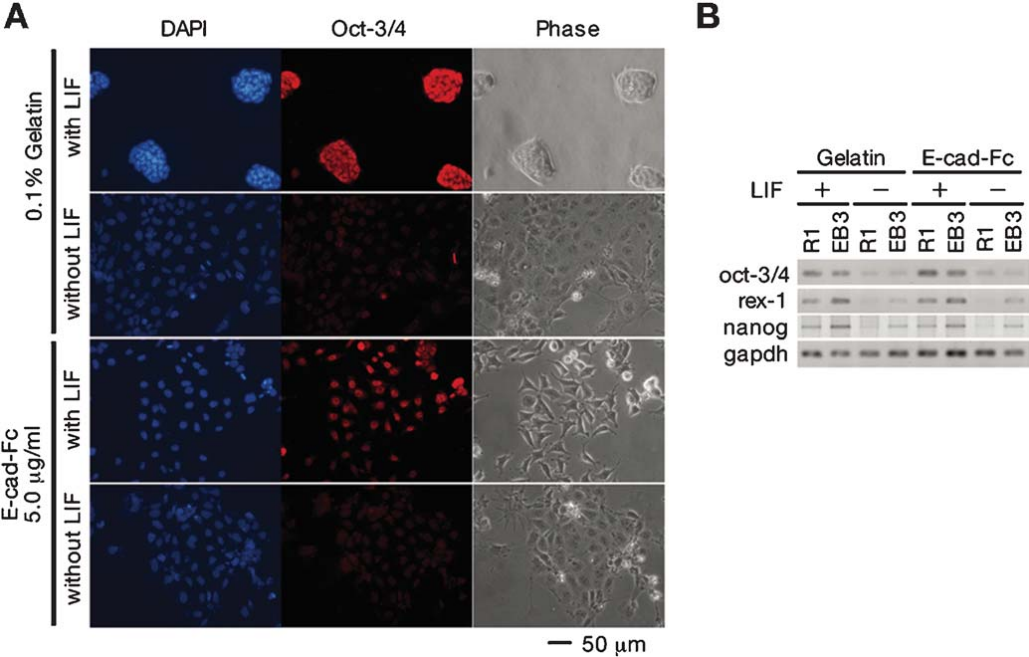
ES cells maintain the undifferentiated phenotype on E-cad-Fc-coated plates. (A) R1 cells cultured for 5 days in the presence or absence of LIF were stained with anti-Oct-3/4-specific antibody. In the presence of LIF, there was nuclear staining of Oct-3/4 in cells cultured on both gelatinized plates or E-cad-Fc-coated plates. (B) The expression of 3 genes that are markers of the undifferentiated state was analyzed by RT-PCR.

When ES cells differentiate, they migrate out from colonies on gelatin-coated dishes, similar to the ES cells on the E-cad-Fc-coated dishes, suggesting that these cells may have differentiated, even in the presence of LIF. To check this point, we examined the expression of Oct-3/4 [16], *Zfp42/rex-1* [17] and *nanog* [18], [19] well-known markers for undifferentiated ES cells. Immunostaining showed that ES cells cultured on E-cad-Fc-coated dishes in the presence of LIF for 5 days expressed Oct-3/4 in the nucleus, and that depletion of LIF led to a marked reduction in Oct-3/4 staining (Figure 2A). The expression of Oct-3/4 was maintained for at least 30 days when cultured on E-cad-Fc-coated dishes. RT-PCR showed that EB3 and R1 cells on both the gelatin-coated and E-cad-Fc-coated dishes expressed the *oct-3/4, rex-1* and *nanog* genes after 14 days culture with LIF supplementation, and that expression of all 3 genes decreased upon LIF withdrawal (Figure 2B). Furthermore, the removal of LIF did not lead to any specific differentiation on the E-cad-Fc-coated surface, indicating that the adhesion onto E-cadherin did not affect the early differentiation of ES cells (data not shown). Alkaline phosphatase (ALP) activity is another marker of the undifferentiated state. There was no apparent difference in ALP activity between ES cells grown on the E-cad-Fc-coated dishes and those on the gelatin-coated dishes (data not shown) in the presence of LIF, indicating that binding to E-cad-Fc did not induce differentiation of the ES cells.

**Figure 3:**
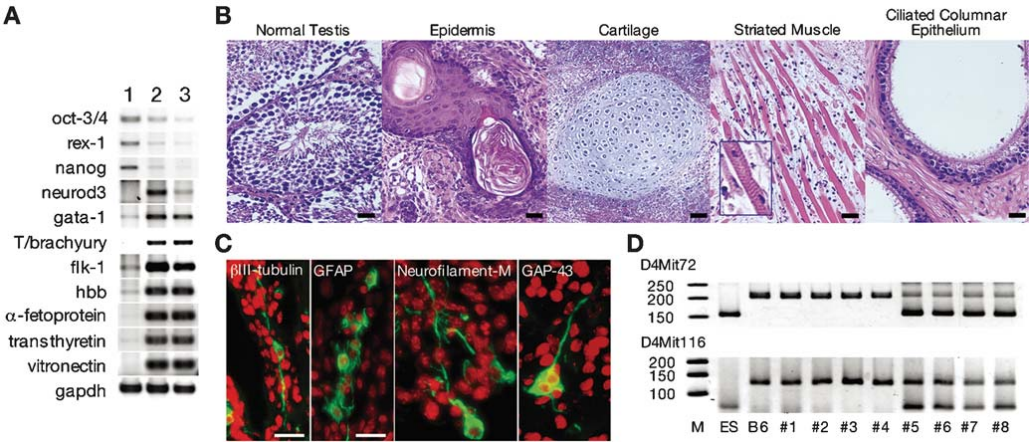
Pluripotency of ES cells on E-cad-Fc-coated surface. (A) R1 cells were maintained on gelatin or E-cad-Fc for 26 days, and then were cultured to form embryoid bodies. After 14 days culture of embryoid bodies, expression of marker genes was analyzed by RT-PCR. Lane 1: undifferentiated cells; lane 2: on gelatin; lane 3: on E-cad-Fc. (B and C) Characterization of teratomas from ES cells (EB3) cultured on an E-cad-Fc-coated surface. (B) H&E staining of teratomas showed the differentiation into ectoderm (epidermis), mesoderm (cartilage, and striated muscle cells) and endoderm (ciliated columnar epithelium, possibly bronchial epithelium). Differentiation into ectoderm was confirmed by specific staining for the neural markers βIII-tubulin, GFAP, Neurofilament-M and GAP-43 (C). Scale bar indicates 50 µm. (D) EB3 cells cultured on gelatin- or E-cad-Fc-coated dishes for 15 days were introduced into approximately 100 blastocysts of C57BL/6 (B6) mice in each group, which yielded 4 and 7 heads of chimera pups, respectively. Furthermore, by mating with wild-type B6 females, 2/4 chimera males from the gelatin-coated group and 3/5 chimera males from the E-cad-Fc-treated group produced offspring with ES cell-derived coat colors, suggesting comparable chimera formation and germ-line transmission abilities in E-cad-Fc-treated ES cells. Germ-line transmission was also verified genetically by DNA microsatellite marker analysis. PCR-based microsatellite marker analysis was performed on a litter mate. The primer sequences for D4Mit72 and D4Mit116 microsatellite markers were obtained from Mouse Microsatellite Data Base of Japan (http://shigen.lab.nig.ac.jp/mouse/mmdbj/top.jsp).

Next, we checked whether the pluripotency of ES cells was affected by culturing on E-cad-Fc-coated dishes. To assess the potential of ES cells to differentiate into multiple cell lineages, embryoid bodies were generated from ES cells that had been cultured and passaged on gelatin or E-cad-Fc in the presence of LIF for 26 days. These embryoid bodies were cultured for an additional 14 days without LIF to induce differentiation. A comparable number of embryoid bodies were obtained from cells cultured on either gelatin or E-cad-Fc. The level of expression of lineage-specific marker genes was analyzed by RT-PCR. Embryoid bodies derived from gelatinized or E-cad-Fc-coated surfaces similarly developed into ectoderm (*neurod3/ngn1*), mesoderm (*gata-1, T/brachyury, flk-1*, and *hbb*) and endoderm (*α-fetoprotein, transthyretin*, and *vitronectin*) derivatives (Figure 3A). Furthermore, the ability for in vitro differentiation to somatic cell types such as neuronal cells [20] and cardiomyocytes [21] was confirmed by well-established culture systems using feeder layers (data not shown).

We also examined the ability of ES cells cultured on E-cad-Fc-immobilized surfaces to form teratomas. ES cells were cultured for 15 days on E-cad-Fc-coated dishes in the presence of LIF and then transplanted into murine testis. Sixty days after transplantation, teratomas were generated that consisted of all three germ layer–derived tissues (Figure 3B,C). As a final confirmation of the maintenance of pluripotency of ES cells grown on E-cad-Fc-coated plates, we generated chimera mice to check the germ-line transmission ability. ES cells cultured on gelatin- or E-cad-Fc-coated dishes for 15 days were introduced into approximately 100 blastocysts in each group, which yielded 4 and 7 heads of chimera pups, respectively. Furthermore, by mating with wild-type females, 2/4 chimera males from the gelatin-coated group and 3/5 chimera males from the E-cad-Fc-treated group produced offspring with ES cell-derived coat colors, suggesting comparable chimera formation and germ-line transmission abilities in E-cad-Fc-treated ES cells. Germ-line transmission was also verified genetically by DNA microsatellite marker analysis. The offspring derived from the chimeric male exhibited ES cell-specific microsatellite patterns (Figure 3D). These results revealed that ES cells cultured on E-cad-Fc-treated plates could maintain their pluripotency.

**Figure 4:**
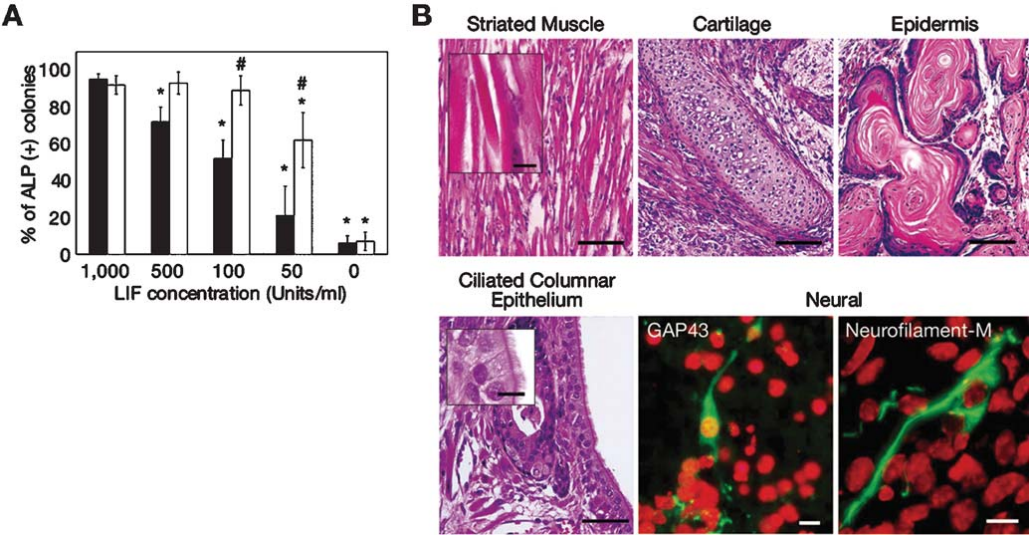
Effect of LIF concentration on the maintenance of undifferentiated state on ES cells. (A) R1 cells were cultured for 5 days in the presence of various doses of LIF (0–1,000 units/ml), on E-cad-Fc-coated plates (open bars) or gelatin-coated plates (closed bars). Then cells were replated onto gelatin-coated plates and three days later, the ratios of ES cell colonies with high ALP activity were estimated. *:*P*<0.05 vs. gelatinized plate in the presence of 1,000 units/ml LIF. #:*P*<0.05 for ES cells cultured on E-cad-Fc-coated plates versus gelatinized plate in the presence of same concentration of LIF. (B) The maintenance of the pluripotent efficiency of ES cells, which was cultured on an E-cad-Fc-coated surface at a low concentration of LIF (100 units/ml), was assessed by the characterisation of teratomas. ES (EB3) cells were maintained on E-cad-Fc-coated surface in the presence of 100 units/ml of LIF and then transplanted into mouse testis. H&E staining of teratomas showed the differentiation into ectoderm (epidermis: top right, bar: 100 µm), mesoderm (striated muscle cells: top left, and cartilage: top centre, bar: 100 µm, inset: 10 µm) and endoderm (ciliated columnar epithelium, possibly bronchial epithelium: bottom left, bar: 50 µm, inset: 10 µm). Differentiation into ectoderm was confirmed by specific staining for the neural markers GAP-43 (bottom centre) and Neurofilament-M (bottom right).

We then examined the dependency of ES cells on E-cad-Fc-coated plates on LIF to maintain an undifferentiated phenotype. On gelatin-coated plates, ALP activity in the ES cells gradually diminished as the concentration of LIF decreased. In contrast, the majority of ES cells on E-cad-Fc-coated dishes retained high levels of ALP activity even when the LIF concentration was decreased 10-fold (Figure 4A). Furthermore, ES cells maintained on an E-cad-Fc-coated surface even at a low LIF condition (100 units/ml) could also form teratomas consisting of three germ layers (Figure 4B). These results demonstrate that the E-cad-Fc culture method can maintain the undifferentiated state and pluripotency of ES cells at markedly lower concentrations of LIF compared with the conventional culture methods.

### ES cells show rapid proliferation and high transfection efficiency on the E-cad-Fc-coated surface

**Figure 5:**
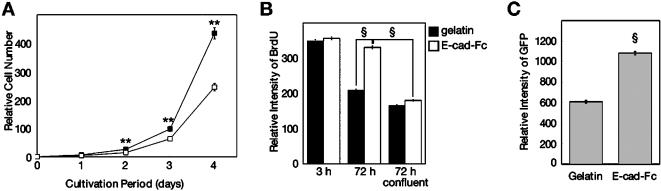
ES cells show higher proliferation and higher transfection efficiency on the E-cad-Fc-coated surface. (A) The proliferative activity of ES cells on a gelatin- or E-cad-Fc-coated surface was evaluated. EB3 cells were seeded on gelatin-coated (open square) or E-cad-Fc-coated (filled square) dishes and the cell number was counted after staining with alamar Blue reagent. The data indicate means±SD of experiments (n = 3). **:*P*<0.01 versus gelatinized plates. (B) BrdU incorporation of EB3 cells under colony-forming (on gelatin) or scattering conditions (on E-cad-Fc). Relative BrdU incorporation value was evaluated. The data indicate means±SEM. §:*P*<0.001. (C) Transfection efficiency of ES3 cells cultured on gelatin- or E-cad-Fc-coated surface. Relative expression of GFP was evaluated. The data indicate means±SEM. §:*P*<0.001 versus gelatinized plates.

The growth curve of the EB3 cells on the E-cad-Fc-coated surface showed an approximately 1.8-fold increase over those on the gelatin-coated dishes (Figure 5A). BrdU uptake experiments showed that EB3 cells grown on this model protein had a mitotic activity that was approximately 1.5–fold higher than that of cells grown on gelatin-coated plates, while there were no significant differences when the assay was carried out after a 3-h culture. Furthermore, the proliferative activity was drastically inhibited by the confluent condition even though cells were cultured as a monolayer on an E-cad-Fc-coated surface (Figure 5B). These results indicated that the packed cell-cell interactions induced by the high cell density inhibited the proliferation of ES cells. Since the colony-forming conditions might also inhibit homogeneous cell access to the transfection liposome, we next investigated the transfection efficiency into ES cells. EB3 cells showed higher expression of GFP when grown under the scattering conditions of the E-cad-Fc-coated surface than those grown under the colony-forming conditions (Figure 5C). R1 cells also showed higher proliferative ability and higher expression of GFP on the E-cad-Fc-coated surface than on the gelatinized surface (data not shown).

## Discussion

We demonstrated that mouse ES cells cultured on E-cad-Fc-coated surface could be maintained with unique morphological character and complete ES cell features, and that they showed higher proliferative ability and transfection efficiency than those grown under conventional conditions. Furthermore, they require less LIF, probably due to the homogeneous exposure to LIF that was achieved in this culture system. We also found that cell growth is inhibited when seeded at high density even on E-cadherin-coated surface. Moreover, Cui et al. reported the heterogeneous distribution of cell-cell adhesion molecules in undifferentiated ES cell colonies [22]. These observations indicate that an aggregated colony formation with close cell-cell communications may generate a heterogeneous environment within the colonies, which potentially inhibit the proliferation of ES cells and the distribution of soluble factors.

Thomson et al. successfully established human ES cell lines from blastocysts [23], and these are potentially a major source of cells for regenerative medicine. However, for therapeutic application, feeder cells, non-human-derived serum and other culture components may be a source of pathogens such as retroviruses. A number of new culture methods for ES cells have recently been developed that do not require feeder cells or serum [24]–[31], but a drawback of these methods is that the undifferentiated ES cells form aggregates, which have a potency to inhibit diffusion of soluble factors to cells, and thereby may affect the pluripotency of the cultured cells. Furthermore, close contact between cells in these aggregates may lead to paracrine interactions with neighbouring cells that could generate heterogeneity and initiate differentiation. Therefore, the application of this system to primate ES cells could be expected to improve the difficulty in culture of primate ES cells.

There are many cell surface proteins that regulate cell proliferation and differentiation. For example, several reports suggested that ephrin-eph signalling inhibit proliferation of cells by preventing the activation of Ras/MAPK signalling cascades [32], [33]. Since ES cells express ephrin and eph receptor depending on the expression of E-cadherin [34], eprin-eph signalling could be activated within the aggregated colonies. On E-cad-Fc-coated surface, most cells were existed as a single cell and cell-cell contact was not detected, causing the drastic reduction of cell-cell communications including ephrin-eph interaction. When cells were seeded at high density on E-cad-Fc, cell-cell communication should be increased. In this condition, proliferation of ES cells was inhibited (Figure 5B). These observations suggested that cell proliferation was inhibited by complicated cell-cell communications.

Moreover, the analysis of signal transduction on a discrete single cell is still difficult or impossible under the colony-forming conditions because intercellular cross talk cannot be discounted. Thus, the new culture system presented here does not pose the same problems as cells can be maintained singly, which is beneficial for the investigation of signalling pathways and cell responses to growth factors, cytokines, and drugs using single cell-based assays. Importantly, the establishment of an efficient mouse ES cell culture system will allow investigation of many physiological mechanisms in development and cell behaviour in vivo or in vitro.

## Materials and Methods

### 

#### Cell culture

For all cultures, feeder-free murine ES cells (EB3, R1) were used. EB3 cells or R1 cells were maintained on 0.1% gelatin-coated surfaces in KNOCKOUT-DMEM (Invitrogen), supplemented with 1 mM L-glutamine, 1% nonessential amino acids (Invitrogen), 0.1 mM β-mercaptoethanol (Sigma Chemical), 10 or 20% (vol/vol) foetal bovine serum (FBS) and 1,000 or 5,000 units/ml ESGRO (Chemicon), respectively. Cells were passaged every 2 or 3 days with 0.25% trypsin-1.0 mM EDTA solution (Invitrogen). All media contained 50 µg/ml penicillin, 50 µg/ml streptomycin, and 100 µg/ml neomycin.

#### Expression of fusion protein (E-cad-Fc) and preparation of E-cad-Fc-coated dishes

Expression and purification of E-cad-Fc fusion proteins were described previously [13]. In brief, the E-cadherin extracellular domain cDNA, which was generated from mouse E-cadherin full-length cDNA provided by the RIKEN BRC DNA Bank (RDB: 1184), and mutated mouse IgG1 Fc domain cDNA (T252M-T254S), which have high affinity to Protein A, were ligated with pRC/CMV (Invitrogen) fragment, which was digested with Hind III and Xba I to generate the expression vector “pRC-ECFC”. CHO-K1 cells were transfected with “pRC-ECFC” using Lipofectamine reagent (Invitrogen) according to the manufacturer's directions. After selection of a highly expressing clone, 4G7 with 400 µg/ml G418 (Invitrogen), conditioned media were collected. The fusion proteins were loaded onto a rProtein A FF column (Amersham Biosciences). The column was washed with 20 mM phosphate buffer (pH 7.0), and the bound proteins were eluted using 0.1 M sodium citrate (pH 2.7) followed by neutralisation with a 1/5 volume of 1.0 M Tris-HCl (pH 9.0). Eluates were dialysed against PBS containing 0.9 mM CaCl_2_ and 0.9 mM MgCl_2_ for 3 days.

To prepare the E-cad-Fc-coated surface, purified E-cad-Fc solution was directly added to non-treated polystyrene plates. After 2 hours incubation at 37°C, plates were washed with PBS once, and then cells were seeded.

#### Adhesion and growth assays

Cells were seeded at a density of 3.0×10^4^ cells/well into 96-well plates precoated with gelatin or E-cad-Fc. After 4 h of culture, medium and non-adherent cells were removed, and cells were washed with culture medium. Adherent cells were stained with alamar Blue reagent (Biosource) and absorbance at 570 nm was measured using a microplate reader. For detachment assays, cells were seeded onto fibronectin- or E-cad-Fc-coated plates and incubated with 5 mM EGTA for 30 min. Remaining cells were counted as mentioned above. For the cell growth assay, cells were seeded at a density of 500 cells/well into a 96-well plate coated with E-cad-Fc or gelatin. The cell number was evaluated once per day.

#### BrdU (5-bromo-2′-deoxyuridine) incorporation and proliferation assay

Cells were seeded onto 24-well plates precoated with gelatin or E-cad-Fc. Three days later, 10 µM BrdU was added and incubated for 30 min. Cells were detached and reseeded onto black-sided 96-well plates (NUNC) coated with E-cad-Fc to analyze as single cells by the ArrayScan™ System (Cellomics). We confirmed that equal numbers of cells adhered to the plates. After a 4 h incubation, medium and non-adherent cells were removed, and cells were fixed in 200 µl of FixDenat reagent (Roche Applied Science) for 25 min at room temperature. After washing with PBS, cells were incubated with Image-iT FX signal enhancer (Invitrogen) for 30 min at room temperature. Incorporated BrdU was stained with an anti-BrdU monoclonal antibody Fab fragment (clone BMG 6H8; Roche Applied Science) followed by Cy3-conjugated anti-mouse IgG F(ab′)_2_ antibody (Jackson Immunoresearch Laboratories). Nuclei were counterstained with 10 µg/ml DAPI (4′, 6-diamidino-2-phenylindole; Sigma Chemical) to identify discrete cells. BrdU incorporation on each individual cell was measured by ArrayScan™ system and the average fluorescence intensity of Cy3 was defined as the relative incorporation of BrdU in discrete cells.

#### Analysis of transfection efficiency

Cells were seeded onto 24-well plates precoated with gelatin or E-cad-Fc. Three days later, pEGFP-N2 plasmid (expression vector for enhanced green fluorescent protein: Clontech) was transfected into the cells using Lipofectamine 2000 reagent (Invitrogen) according to the manufacturer's instructions. One day after transfection, cells were detached and reseeded onto black-sided 96-well plate (NUNC) coated with E-cad-Fc to analyze as single cells by the ArrayScan™ System (Cellomics). After a 4-h incubation, medium and non-adherent cells were removed, and cells were fixed with 8% formaldehyde solution (pH 7.0–7.5; Wako Pure Chemical) for 10 min and permeabilised with 0.2% Triton X-100 for 2 min at room temperature. Cells were incubated with Image-iT FX signal enhancer (Invitrogen) for 30 min at room temperature. GFP was stained with anti-GFP monoclonal antibody (Nacalai Tesque) followed by Alexa Fluor 546-conjugated secondary antibody (Invitrogen). Nuclei were counterstained with 1.0 µg/ml DAPI to identify discrete cells. The level of GFP expression on each individual cells was measured by ArrayScan™ system and the average fluorescence intensity of Alexa Fluor-546 was defined as the relative expression of GFP in discrete cells.

#### Teratoma formation assay

Balb/c nude mice (n = 5, each) were purchased from Japan CLEA (Tokyo, Japan). Control ES cells grown on gelatin-coated dishes and the ES cells that were cultured and passaged 5 times on E-cad-Fc-coated dishes were transplanted into the right testis of mice (200 cells/mouse). The left testis was used as the non-transplanted control. All experimental procedures and protocols were reviewed and approved by the Animal Care and Use Committees of the Keio University and conformed to the NIH Guide for the Care and Use of Laboratory Animals

#### Histological analysis, ALP staining and immunofluorescence

For immunofluorescence staining, cells were fixed with 8% formaldehyde solution (pH 7.0–7.5; Wako Pure Chemical) for 10 min and permeabilised with 0.2% Triton X-100 for 2 min at room temperature. Fixed cells were incubated with Image-iT FX signal enhancer (Invitrogen) for 30 min at room temperature. Oct-3/4 was stained with an anti-mouse Oct-3/4 polyclonal antibody (H-134; Santa Cruz Biotechnology) for 2 h followed by Alexa Fluor 546-conjugated secondary antibody (Invitrogen) for 1 h. Nuclei were counterstained with 0.5 µg/ml DAPI. Samples were observed by fluorescence microscopy. For analysis of teratomas, the mice were sacrificed at 60 days after transplantation. The testis was dissected, and was cut into 2 pieces. One was fixed with 10% formalin, embedded in paraffin, and sectioned into 4-µm-thick slices for hematoxylin-eosin staining. For immunostaining, the other part was embedded into OCT compound and frozen with liquid nitrogen, and then sectioned into 6-µm-thick slices using a cryostat. Non-specific binding of antibodies was blocked by incubation with 4% BSA/PBS for 30 min at room temperature. The samples were exposed to the primary antibodies, anti-βIII-tubulin mouse monoclonal antibody (1∶100, Promega), anti-GFAP rabbit polyclonal antibody (1∶100, Dako), anti-Neurofilament-M rabbit polyclonal antibody (1∶100, Chemicon), or anti-GAP43 rabbit polyclonal antibody (1∶100, Chemicon) overnight at 4°C, followed by incubation with the secondary antibody conjugated with Alexa Fluor 488 (Invitrogen) for 30 min at room temperature. The samples were washed twice with PBS, treated with 0.5 µg/ml Toto-3 (Sigma Chemical) for 2 min, and were observed by confocal LASER microscopy (LSM510, Carl Zeiss International). Alkaline phosphatase activity was determined using a Sigma Diagnostics Alkaline phosphatase kit (Sigma Chemical).

#### RT-PCR analysis

Total RNA was isolated with Trizol reagent (Invitrogen). First strand cDNA was synthesized using Moloney murine leukemia virus (M-MLV) reverse transcriptase (Invitrogen), and PCR was carried out with rTaq polymerase (TOYOBO) in the reaction buffer containing 1.5 mM MgCl_2_. Primers used for *oct-3/4, rex-1, nanog, neurod3/ngn1, gata-1, T/brachyury, flk-1, hbb, α-fetoprotein, transthyretin, vitronectin*, and* gapdh* are listed in Table S1. PCR products were analyzed by 2% agarose gel electrophoresis.

#### Statistical Analysis

Values are reported as means±SD or SEM, and statistical significance was assessed using the paired Student's t-test. The probability level accepted for significance was *P*<0.05.

## Supporting Information

Table S1 Primer sequences used in this study.
(0.03 MB PDF)

Movie S1 ES cells (EB3) were cultured on 0.1% gelatin coated dishes. The cells form aggregations and do not move out from the colonies.
(2.67 MB MOV)

Movie S2 ES cells (EB3) were cultured on E-cad-Fc-coated dishes. Each cell moved freely over the dishes with pseudopodial protrusions like amoeba.
(2.73 MB MOV)
