# Supplementary material for: E-Cadherin-Coated Plates Maintain Pluripotent ES Cells without Colony Formation
Source: PLoS One. 2006 Dec 20;1(1):e15. doi: 10.1371/journal.pone.0000015 (PMC1762325; doi:10.1371/journal.pone.0000015)
Supplement: Table S1 — Primer sequences used in this study. (0.03 MB PDF) [file pone.0000015.s001.pdf]

Gene (Unigene Symbol)	5' primer (5' to 3')	3' primer (5' to 3')	product length (bp)	annealing temp (°C)	cycle numbers
oct-3/4 (Mm.17031)	GAAGTTGGAGAAGGTGGAACC	GCCTCATACTCTTCTCCGTTGG	528	60	20
Zfp42/rex-1 (Mm.285848)	AAAGTGAGATTAGCCCCGAG	TCCCATCCCCTTCAATAGCA	930	60	20
nanog (Mm.6047)	GAGGAAGCATCGAATTCTGG	AAGTTATGGAGCGGAGCAGC	710	60	18
neurod3/ngn1 (Mm.266665)	CATCTCTGATCTCGACTGC	CCAGATGTAGTTGTAGGCG	405	59	30
gata-1 (Mm.335973)	TTGGACACCTTGAAGACGG	GCATAAGATGGCTGACAGGC	745	60	26
T/brachyury (Mm.913)	TGCTGCCTGTGAGTCATAAC	TCCAGGTGCTATATATTGCC	947	59	30
flk-1 (Mm.285)	TAGGTGCCTCCCCATACCCTGG	TGGCCGGCTCTTTCGCTTACTG	398	60	26
hbb (Mm.288567)	AACCCTCAATGGCCTGTGG	TCAGTGGTACTTGTGGGACAGC	415	60	26
α*-fetoprotein* (Mm.358570)	GCTCACACCAAAGCGTCAAC	CCTGTGAACTCTGGTATCAG	410	60	26
transthyretin (Mm.2108)	AGTCCTGGATGCTGTCCGAG	TTCCTGAGCTGCTAACACGG	440	60	26
vitronectin (Mm.3667)	CTGCGAGCCCATTCAGAGCG	TCAGAGGTCGGGCAGCCCAGC	141	60	26
gapdh (Mm.333399)	GGAAGCTTGTCATCAACGG	CTCTTGCTCAGTGTCCTTGC	858	60	20

Table S1. PCR primers used in this study
